# Procyanidin C1 Activates the Nrf2/HO-1 Signaling Pathway to Prevent Glutamate-Induced Apoptotic HT22 Cell Death

**DOI:** 10.3390/ijms20010142

**Published:** 2019-01-02

**Authors:** Ji Hoon Song, Hae-Jeung Lee, Ki Sung Kang

**Affiliations:** 1 College of Korean Medicine, Gachon University, Seongnam 13120, Korea; jhsong.john@gmail.com; 2 College of Bio-Nano Technology, Department of Food and Nutrition, Gachon University, Seongnam 13120, Korea

**Keywords:** procyanidin C1, glutamate, oxidative stress, Nrf2/HO-1 signaling pathway, MAPK, neuroprotective effects

## Abstract

Natural sources are very promising materials for the discovery of novel bioactive compounds with diverse pharmacological effects. In recent years, many researchers have focused on natural sources as a means to prevent neuronal cell death in neuropathological conditions. This study focused on identifying neuroprotective compounds and their underlying molecular mechanisms. Procyanidin C1 (PC-1) was isolated from grape seeds and assessed for biological effects against glutamate-induced HT22 cell death. The results showed that PC-1 strongly prevented glutamate-induced HT22 cell death. Moreover, PC-1 was also found to prevent glutamate-induced chromatin condensation and reduce the number of annexin V-positive cells indicating apoptotic cell death. Procyanidin C1 possessed a strong 2,2-diphenyl-1-picrylhydrazyl (DPPH) radical-scavenging activity and inhibited glutamate-induced accumulation of intracellular reactive oxygen species and protein carbonylation. Additionally, PC-1 mediated nuclear translocation of nuclear factor erythroid-derived 2-related factor 2 and increased the expression levels of heme oxygenase (HO-1). Inhibition of HO-1 by tin protoporphyrin, a synthetic inhibitor, reduced the protective effect of PC-1. Furthermore, PC-1 also blocked glutamate-induced phosphorylation of mitogen-activated protein kinases (MAPKs) including ERK1/2 and p38, but not JNK. This study is the first experimental report to demonstrate the neuroprotective effects of PC-1 against glutamate-induced cytotoxicity in HT22 cells. Therefore, our results suggest that PC-1, as a potent bioactive compound of grape seeds, can prevent neuronal cell death in neuropathological conditions.

## 1. Introduction

The central nervous system is especially vulnerable to oxidative stress because of its high content of polyunsaturated fatty acid, high demand for oxygen consumption, and limited antioxidative systems [1]. Oxidative stress plays a crucial role in neuronal cell death, contributing to neurological diseases including Parkinson’s disease, Alzheimer’s disease, and strokes [2]. It is well known that excessive oxidative stress can cause DNA and protein oxidation and lipid peroxidation and promotes neuronal cell death [3,4]. In addition, oxidative stress can enhance intracellular Ca^2+^ concentration [5], and activate neuro-inflammatory reactions and apoptotic pathways [6]. A previous study has suggested that excessive levels of oxidative stress and the disturbance of the antioxidant defense enzyme system in the brain constitute a pathological biomarker for neurological diseases [7]. Therefore, the prevention of oxidative stress could be a potential therapeutic target for neurological diseases, and many researchers have contributed to the identification of agents which possess antioxidant activity or enhance antioxidant defense enzymes.

In the brain, glutamate is the important excitatory neurotransmitter and is critical in brain development and neuronal cell damage in conjunction with oxidative stress [2]. Glutamate induced neuronal cell death in the presence of its higher levels at extracellular region through both receptor-initiated excitotoxicity and non-receptor-mediated oxidative stress [8]. Immortalized neuronal HT22 cells originating from the mouse hippocampus lack functional ionotropic glutamate receptors [9,10]. Thus, HT22 cells are commonly used to study the mechanisms of neuronal cell death via the non-receptor-mediated oxidative pathway.

There are several antioxidant enzymes, including thioredoxin reductase, glutathione peroxidase, catalase, and heme oxygenase-1 (HO-1), which respond to cellular oxidative stress [4]. Recent studies have demonstrated that the Nrf2 pathway is critical in regulating the expression of these enzymes. Specifically, HO-1 is an inducible antioxidant enzyme which is a major target gene regulated by Nrf2. HO-1 plays an important role in protecting cells against oxidative stress [11,12]. Previous studies have suggested that the induction of HO-1 prevents glutamate-mediated oxidative stress in HT22 cells [13,14]. In addition, mitogen-activated protein kinases (MAPKs), such as ERK1/2, p38, and JNK, also play a crucial role in oxidative stress-mediated neuronal cell death [9,15]. Many studies have shown that glutamate-induced oxidative stress triggers the prolonged activation of MAPKs, resulting in neuronal cell death [15].

Nowadays, herbal extracts and natural products are attractable for their neuroprotective capacity with their antioxidant properties. They can significantly alleviate the symptoms related to diseases caused by oxidative stress. Thus, it is reasonable to exploit effective natural products from antioxidant-rich medicinal plants for the treatment of oxidative damage. Grape seed extracts possess a high number of polyphenols and has shown many biological effects such as anti-cancer, anti-inflammation, and cardioprotection [16]. It has also demonstrated a neuroprotective effect in ischemic brain injury [17]. Procyanidin C-1 (PC-1) is known as a phenolic compound in grape seed extract and shows a strong antioxidant activity [18]. However, the protective effects of PC-1 on neuronal cell death and its protective mechanisms have not been reported yet. Therefore, the present study investigated the neuroprotective effects of PC-1, a potential active compound from grape seed extracts, using the murine hippocampal cell line, HT22 cells.

## 2. Results and Discussion

### 2.1. Neuroprotective Effects of PC-1

**Figure 1:**
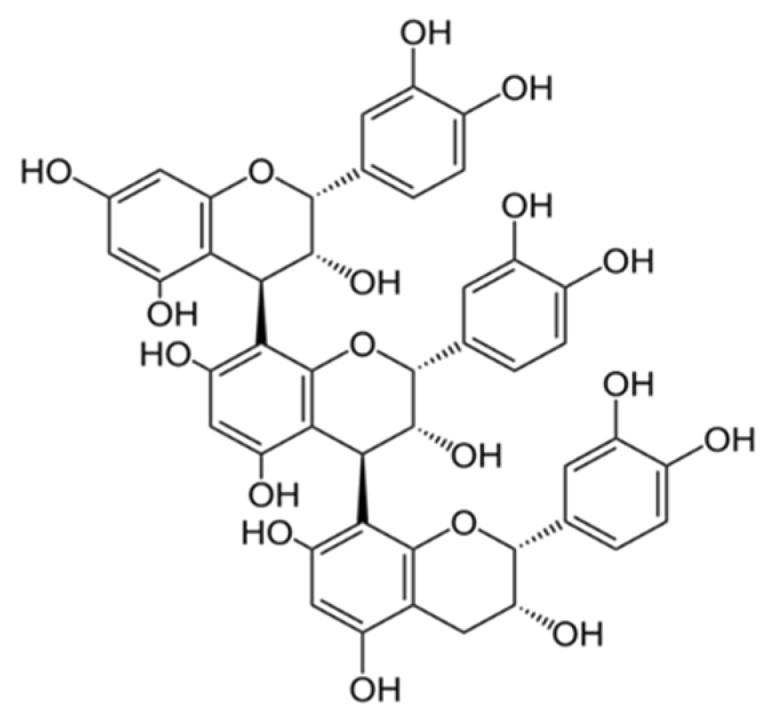
The chemical structure of procyanidin C1 (PC-1).

**Figure 2:**
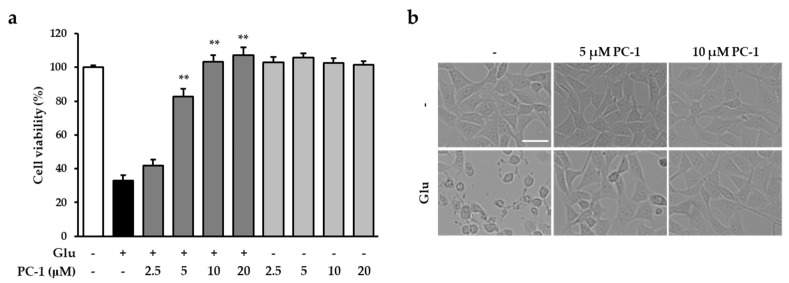
Neuroprotective effects of PC-1 isolated from grape seed in HT22 cells. (**a**) The cells were treated with 5 mM glutamate and the indicated concentrations of PC-1. Data are presented as the mean value ± S.E.M. ** *p* < 0.001 versus glutamate-treated HT22 cells. (**b**) Representative microscopic images were obtained. Scale bar, 50 μm.

Under neuropathological conditions, the concentration of glutamate is excessively high in extracellular regions. Previously, the existence of high glutamate-induced neuronal cell death in in vitro and in vivo models has been confirmed in both neurodegenerative diseases and acute brain injuries [19]. An immortalized mouse hippocampal cell line, HT22 cells are commonly used for investigating the mechanism of glutamate-induced neurotoxicity [10]. For this reason, we used HT22 cells to identify neuroprotective compounds and study their underlying molecular mechanisms. Using this cell line, we recently reported that ellagitannins from natural plant, such as casuarinin and chebulinic acid, strongly prevented glutamate-induced HT22 cell death neuroprotective effects [20,21]. Therefore, in this study, we examined whether PC-1 (Figure 1) from grape seeds can protect HT22 cells from glutamate-induced cytotoxicity. In this study, HT22 cells were exposed to 5 mM glutamate in the presence of PC-1 for 24 h to assess its neuroprotective effects. As a result, PC-1 above 5 μM significantly reduced neuronal cell death induced by glutamate (Figure 2a). The HT22 cells treated with 5 and 10 μM PC-1 shared a similar morphology with the normal control cells compared to glutamate-treated cells (Figure 2b). These results suggest that PC-1 from grape seeds could be a valuable compound for exhibiting neuroprotective effects. We further investigated the protective mechanism of PC-1 against glutamate-induced HT22 cell death.

### 2.2. Preventive Effect of PC-1 against Glutamate-Induced Apoptosis in HT22 Cells

**Figure 3:**
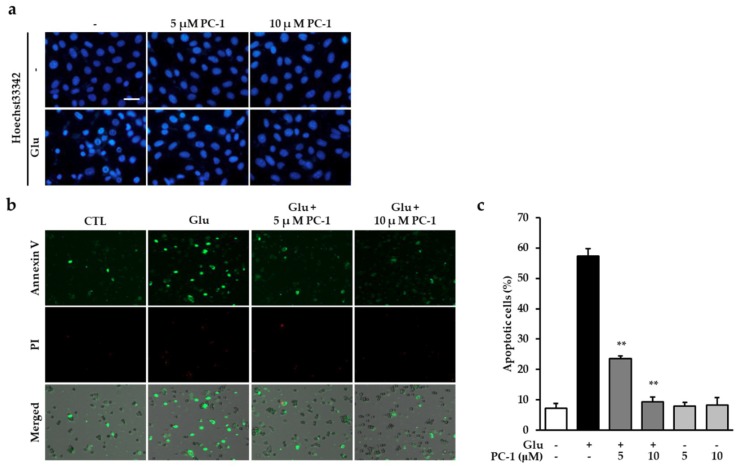
Procyanidin C1 prevented glutamate-induced apoptosis in HT22 cells. (**a**) After 12 h exposure to 5 mM glutamate in the presence or absence of 5 or 10 μM PC-1, the nuclei were visualized with Hoechst 33342. Fluorescent images were acquired using a fluorescent microscope. Scale bar, 20 μm. (**b**) HT22 cells were exposed to 5 mM glutamate in the presence of 5 or 10 μM PC-1 for 10 h and stained with Alexa Fluor 488-conjugated annexin V and PI to evaluate the number of apoptotic and dead cells, respectively. (**c**) Images were quantitatively analyzed using TaliPCApp software. Bars denote the percentage of annexin V-positive cells (apoptotic cells). Data are presented as the mean value ± S.E.M. ** *p* < 0.001 versus glutamate-treated HT22 cells.

Glutamate induces neuronal cell death via both necrotic and apoptotic pathways. Previously, in HT22 cells, glutamate was found to induce necrosis relatively quickly, whereas the majority of cells were apoptotic at later stages [9,10]. In this study, we focused on preventing glutamate-induced apoptosis in HT22 cells. To evaluate the preventive effect of PC-1 against glutamate-induced apoptosis, we first examined chromatin condensation, a key characteristic of apoptotic cell death. Our results indicated that treatment with glutamate increased chromatin condensation in HT22 cells, while PC-1 levels markedly diminished (Figure 3a). We further performed an image-based cytometric analysis to quantify the proportion of apoptotic cells in each group. The HT22 cells were exposed to 5 mM glutamate in the presence or absence of PC-1 for 12 h and labeled with Alexa Fluor 488-conjugated annexin V and propidium iodide (PI). The resulting percentage of annexin V-positive apoptotic cells was 57.4% after treatment with glutamate, while PC-1 significantly reduced the percentage of apoptotic cells to 23.5 and 9.31% after treatment with 5 and 10 μM PC-1, respectively (Figure 3b,c). Representative images indicate that the majority of cells treated with glutamate were annexin V-positive (apoptotic) cells, which were reduced by PC-1, and only a few PI-positive cells were detected (Figure 3c). These results suggest that the protective effect of PC-1 against glutamate-induced HT22 cell death is due to its anti-apoptotic properties.

### 2.3. The Effects of PC-1 on Glutamate-Induced Oxidative Stress

It is well known that the level of intracellular reactive oxygen species (ROS) is tightly regulated by an intracellular antioxidant defense system. However, oxidative stress is caused by excessive accumulation of intracellular ROS due to the disruption of the balance between the production of ROS and antioxidant activity. Oxidative stress is commonly known as a causative factor for neuronal cell death in neuropathological conditions. High concentrations of glutamate trigger oxidative stress which in turn contribute to neuronal cell death in neurodegenerative diseases and acute brain injuries. The literature suggests that the prevention of oxidative stress is a powerful tool for protecting neurons.

**Figure 4:**
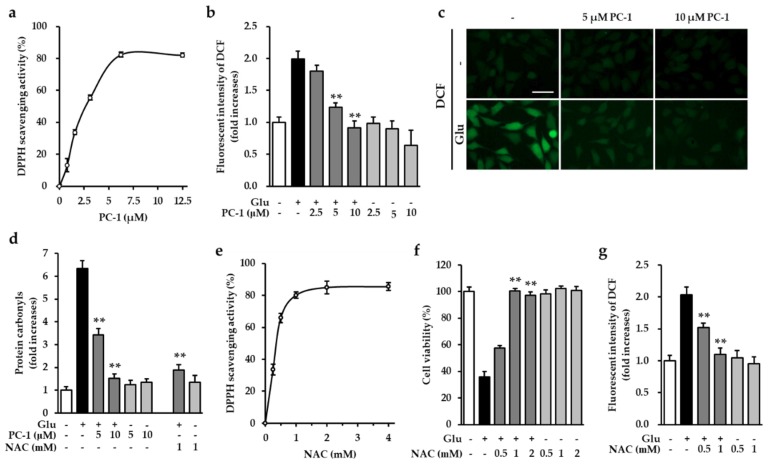
Procyanidin C1 prevented glutamate-induced oxidative stress in HT22 cells. (**a**) We assayed antioxidant activity using a 2,2-diphenyl-1-picrylhydrazyl (DPPH) radical-scavenging activity assay. Lines indicate the percentage of DPPH scavenging activity. (**b**) HT22 cells were exposed to 5 mM glutamate in the presence or absence of 5 or 10 μM PC-1 for 8 h and stained with H_2_DCFDA. The fluorescent intensity of DCF was measured using a fluorescent microplate reader. Bars denote the fold-increase of intracellular reactive oxygen species (ROS) levels. Data were represented by the mean value ± S.E.M. ** *p* < 0.001 versus glutamate-treated HT22 cells. (**c**) Representative fluorescent images were obtained using a fluorescent microscopy. Scale bar, 50 μm. (**d**) HT22 cells were incubated for 8 h with 5 mM glutamate in the presence or absence of PC-1 or N-acetyl-cysteine (NAC) and determined protein oxidation (mean ± S.E.M. ** *p* < 0.001 versus glutamate-treated HT22 cells). (**e**) Lines indicate the percentage of DPPH scavenging activity of NAC, as a positive control. (**f**) As a positive control, NAC was tested for neuroprotective effects on glutamate-induced HT22 cell death (mean ± S.E.M. ** *p* < 0.001 versus glutamate-treated HT22 cells). (**g**) HT22 cells were exposed to 5 mM glutamate in the presence or absence of 0.5 or 1 mM NAC for 8 h and stained with H_2_DCFDA. The fluorescent intensity of DCF was measured using a fluorescent microplate reader. Bars denote the fold-increase of intracellular ROS levels.

Glutamate-mediated oxidative stress triggers neuronal death in culture systems, such as primary neuronal cell cultures and cell lines [22]. It has been reported that flavonoids and ellagitannins with strong antioxidant activity prevent glutamate-induced neuronal death [23]. These results indicate that preventing the accumulation of intracellular ROS is a possible strategy for protecting neurons against glutamate-induced cell death. Therefore, we initially tested the antioxidant activities of PC-1 using a 2,2-diphenyl-1-picrylhydrazyl (DPPH) radical-scavenging activity assay. The result showed that PC-1 exhibited strong antioxidant properties, as indicated by DPPH radical scavenging activity (Figure 4a). This suggests that the antioxidant properties of PC-1 can reduce the accumulation of intracellular ROS. We further tested whether PC-1 prevents the glutamate-induced accumulation of intracellular ROS. As shown in Figure 4b, fluorescent images showed that treatment with glutamate triggers increased intracellular ROS levels while PC-1 almost completely blocked this increase (Figure 4b). Consistent with this result, our quantitative results showed that glutamate treatment increased intracellular ROS level (1.99-fold increase) was significantly reduced by 5 and 10 μM PC-1 (1.23- and 0.91-fold increases, respectively) (Figure 4c). Excessive ROS can modify lipids, nucleic acid, and proteins [24]. Protein carbonylation is an attractive marker of oxidative stress and associated with various disease including chronic renal failure, Alzheimer’s disease, and ischemic reperfusion injury [25]. Therefore, we carried out protein oxidation assay to access antioxidant activity of PC-1 against oxidative stress induced by glutamate. Our results showed that PC-1 as well as N-acetyl-cysteine (NAC), known as a strong antioxidant, significantly reduced protein carbonylation by glutamate-triggered oxidative stress (Figure 4d). As a positive control, the treatment with NAC also showed a strong DPPH scavenging activity and preventive effect of glutamate-induced HT22 cell death (Figure 4e,f). N-acetyl-cysteine also blocked the accumulation of intracellular ROS induced by glutamate (Figure 4g). These results suggest that PC-1 prevents glutamate-induced oxidative stress through its antioxidant properties.

### 2.4. Effects of PC1 on Nuclear Translocation of Nrf2 and Expression of HO-1

Heme oxygenase-1 is an important component in the cellular antioxidant system. Heme oxygenase-1 gene expression is inducible, and it has been demonstrated to be involved in protection against glutamate-induced oxidative damage in HT22 cells [26]. Induction of HO-1 expression is performed at the transcriptional level, and its expression is regulated by nuclear factor erythroid 2 like 2 (Nrf2) [27]. This Nrf2 has been reported to induce the expression of various antioxidant stress-related proteins, including glutathione (GSH) and HO-1 [12].

**Figure 5:**
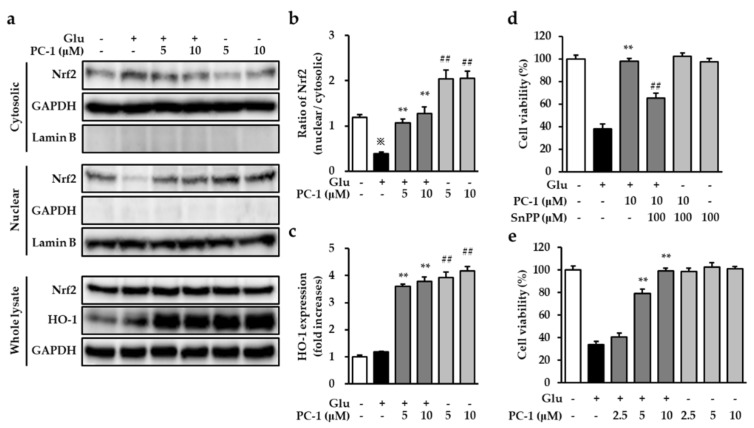
Procyanidin C1 increases the Nrf2/HO-1 pathway in HT22 cells. (**a**) HT22 cells were exposed to 5 mM glutamate in the presence or absence of 5 or 10 μM PC-1 for 6 h and western blot analysis was performed using Nrf2, HO-1, Lamin B, and GAPDH antibodies. The GAPDH and Lamin B were used as markers for cytosolic and nuclear fractions, respectively. (**b**) Bars indicate the ratio of nuclear Nrf2 to cytosolic Nrf2 indicating the nuclear translocation. Data are presented as the mean value ± S.E.M. ※ *p* < 0.001 versus control cells, ** *p* < 0.001 versus glutamate-treated cells, and ## *p* < 0.001 versus control cells. (**c**) Bars denote the fold-increases of HO-1 expression. Data are presented as the mean value ± S.E.M. ** *p* < 0.001 versus glutamate-treated cells, and ## *p* < 0.001 versus control cells. (**d**) The cells were treated with 100 μM SnPP and 10 μM PC-1 followed by the treatment with 5 mM glutamate, and the viability of cells were determined. Data are presented as the mean value ± S.E.M. ** *p* < 0.001 versus glutamate-treated cells and ## *p* < 0.001 versus cells treated with glutamate and PC-1. (**e**) After the pre-treatment with PC-1 for 4 h, cells were treated with 5 mM glutamate for 24 h. Data are presented as the mean value ± S.E.M. ** *p* < 0.001 versus glutamate-treated cells.

Therefore, we examined the effects of PC-1 on the translocation Nrf2 and the expression of HO-1 in HT22 cells. After exposure to 5 mM glutamate in the presence or absence of 5 or 10 μM PC-1 for 6 h, we prepared whole lysate, cytosolic, and nuclear fractions to evaluate the nuclear translocation of Nrf2 and the expression of HO-1. Our results showed that treatment with glutamate significantly decreased the nuclear translocation of Nrf2 compared with the control group. However, nuclear Nrf2 increased significantly by PC-1 treated with or without glutamate in HT22 cells (Figure 5a,b). We also confirmed whether there is any change in the expression of Nrf2 protein. The results indicated that the expression of Nrf2 protein was not changed in all experimental groups (Figure 5a). In addition to this, our results showed that HO-1 expression significantly increased as a result of treatment with PC-1 in the presence or absence of glutamate, whereas treatment of glutamate alone did not affect expression of HO-1 (Figure 5a,c). These results suggest that Nrf2-mediated HO-1 expression might be a possible molecular mechanism for eliminating glutamate-induced oxidative stress by PC-1 in HT22 cells.

In this study, although PC-1 by itself did not induce ROS elevation, PC-1 induced HO-1 expression through triggering nuclear translocation of Nrf2 without any cell death in HT22 cells. This indicated that the increase in the HO-1 expression was not deleterious to HT22 cells. Therefore, we further elucidated whether the increase in the nuclear translocation of Nrf2 and expression of HO-1 by PC-1 contributed to the protection of HT22 cells from glutamate toxicity. To confirm the effect of HO-1, we used a tin protoporphyrin (SnPP), a synthetic inhibitor for HO-1, to block HO-1 activity. As shown in Figure 5d, the presence of 100 μM SnPP did not affect cell viability and partially blocked the protective effect of PC-1 against glutamate-induced HT22 cell death. Additionally, the protective effect of PC-1 did not alter even though cells were pre-treated with PC-1 for 4 h to allow the enough expression of HO-1 (Figure 5e). Taken together, the inhibition of glutamate-induced oxidative stress by PC-1 resulted not only from its antioxidative properties but also from the activation of antioxidant enzymes, such as HO-1. Furthermore, HO-1 activated by PC-1 is effective at the time when ROS has been increased.

### 2.5. Effects of PC-1 on Glutamate-Induced MAPK Activation

**Figure 6:**
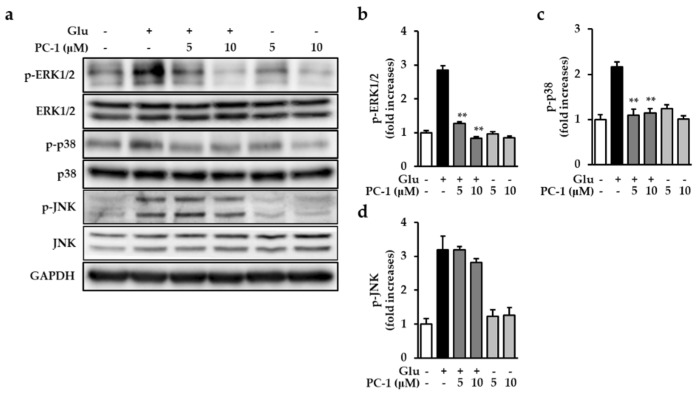
Procyanidin C1 inhibits glutamate-induced MAPK activation in HT22 cells. (**a**) HT22 cells were exposed to 5 mM glutamate in the presence or absence of 5 or 10 μM PC-1 for 8 h, and western blot analysis was performed using antibodies for ERK1/2, p38, and GAPDH. The GAPDH was used for loading control. (**b**–**d**) Bars indicate the fold-increases of ERK1/2, p-38, and JNK phosphorylation compared with the control cells. Data are presented as the mean value ± S.E.M. ** *p* < 0.001 versus glutamate-treated HT22 cells.

Mitogen-activated protein kinase plays a key role in cell survival and death, especially in neuronal cells. During oxidative stress-mediated neuronal cell death, MAPKs, including ERK, p38, and JNK, are excessively phosphorylated and are sustained for a long time [28]. Previous studies suggested that glutamate-induced oxidative stress can trigger prolonged activation of ERK, p38, and JNK, resulting in neuronal cell death [29,30]. This could be a possible molecular mechanism of neuroprotection against glutamate-induced cytotoxicity during neurodegeneration. Therefore, we focused on whether PC-1 could block the phosphorylation of MAPKs induced by glutamate in HT22 cells. To confirm this, HT22 cells were treated with 5 mM glutamate for 8 h in the presence or absence of PC-1 and western blotting analysis was performed. As shown in Figure 6a, our results showed that PC-1 significantly decreased the phosphorylation of ERK1/2 and p38 which were in turn increased by glutamate (Figure 6b,c). However, phosphorylation of JNK increased by glutamate was not decreased by the treatment with PC-1 (Figure 6d). These data suggest that the prevention of oxidative stress by PC-1 can reduce the excessive phosphorylation of MAPKs, specifically in ERK and p38, during glutamate-induced neuronal cell death.

## 3. Materials and Methods

### 3.1. Isolation of PC-1

Procyanidin C1 was isolated from grape seed extract by column chromatography using a Sephadex LH-20 followed by preparative HPLC. The structure of the isolated PC-1 was confirmed by the ESI-MS method. Briefly, 50 mL of aqueous grape seed extract was loaded on a Sephadex LH-20 column, and procyanidin reach fractions were eluted with a 500 mL methanol/water solution (30:70, *v*/*v*). The fraction containing PC-1 was eluted with 500 mL acetone/water solution (70:30, *v*/*v*). The PC-1-rich fraction was further purified by preparative HPLC.

### 3.2. Cell Culture and Treatment

In this study, we used HT22 cells, the immortalized mouse hippocampal cell line. The HT22 cells were cultured in complete growth medium; Dulbecco’s modified Eagle’s medium (DMEM) (Corning, Manassas, VA, USA) supplemented with 10% fetal bovine serum (Atlas, Fort Collins, CO, USA) and antibiotics (penicillin/streptomycin; Gibco, Grand Island, NY, USA). The cells were maintained at 37 °C in a humidified incubator supplied with 5% CO_2_. For the experiments, the cells were plated onto multi-well plates at a density of 2.5 × 10 cells/cm^2^ and incubated for 24 h to adhere before treatment. The cells were then exposed to 5 mM glutamate (Sigma-Aldrich; St. Louis, MO, USA) in the presence or absence of PC-1 for the indicated time.

### 3.3. Assessment of Neuroprotective Effects

The protective effect of PC-1 against glutamate-induced HT22 cell death was assessed using an MTT assay kit (EZ-Cytox; Daeil Lab Service, Seoul, Korea) according to the manufacturer’s instructions. In brief, HT22 cells were plated onto 96-well plates. The cells were treated with glutamate and various concentrations of PC-1. After exposure to glutamate for 24 h, the cells were incubated with 10 µL of EZ-Cytox reagent for 30 min. The absorbance was measured at 450 nm using an E-Max microplate reader (Molecular Devices, Sunnyvale, CA, USA). Cell viability was calculated by the percentage of control cells. In addition, any morphological changes were confirmed using a IX50 fluorescent microscope (Olympus, Tokyo, Japan) equipped with a CCD camera.

### 3.4. Nuclear Staining

To verify the chromatin condensation, HT22 cells were plated onto 6-well plates and stained with Hoechst 33342 (Sigma) [31]. After treatment with glutamate for 12 h, cells were incubated with 10 μM of Hoechst 33342 for 10 min. Fluorescent images were obtained using a fluorescent microscope (IX50) equipped with a CCD camera.

### 3.5. Quantificative Analysis of Apoptotic Cells

The HT22 cells were plated onto 6-well plates and incubated with glutamate and PC-1 (5 or 10 μM) for 12 h. The proportion of apoptotic cells were then quantitatively analyzed using a Tali Image-Based Cytometer (Invitrogen, Eugene, OR, USA) according to the manufacturer’s instructions. After exposure to glutamate for 12 h, cells were harvested and washed with phosphate buffered saline (PBS). The same number of cells were suspended in annexin-binding buffer and labeled with Alexa Fluor 488-conjugated annexin V for 20 min in the dark. The cells were then washed with PBS to remove unbound Alexa Fluor 488-conjugated annexin V followed by staining with PI for 5 min. After washing the cells with PBS, images were acquired using a Tali Image-Based Cytometer and analyzed with the TaliPCApp (version 1.0) program (Invitrogen). Apoptotic cells were represented by the percentage of annexin V-positive cells.

### 3.6. Assessment of Antioxidative Activity of PC-1

The antioxidant activity of PC-1 was assessed using an assay for 1,1-diphenyl-2-picrylhydrazyl (DPPH; Sigma) radical-scavenging activity [32]. Various concentrations of PC-1 were prepared and mixed with an equal volume of DPPH solution. After incubation for 30 min at room temperature, the absorbance was measured using an E-Max microplate reader at a wavelength of 540 nm.

### 3.7. Determination of Intracellular ROS

The HT22 cells were plated on black 96-well plates and exposed to glutamate for 8 h. The cells were stained with 2′,7′-dichlorofluorescin diacetate (H_2_DCFDA; Sigma) to determine the levels of intracellular ROS [33]. After exposure to 5 mM glutamate for 8 h, the cells were incubated with 10 µM H_2_DCFDA for 30 min followed by washing with PBS. The fluorescence intensity of DCF was measured using a microplate reader (SPARK 10M; Tecan, Männedorf, Switzerland) at 495/517 nm (ex/em). Fluorescent intensities were then normalized with those of control cells and represented by fold-increases. Furthermore, fluorescent images were obtained using a fluorescent microscope (IX50; Olympus) equipped with a CCD camera.

### 3.8. Determination of Carbonyl Contents

Fifty microliters of DNPH solution (10 mM) were added into 250 μL of protein samples (1 mg/mL) and vortex-mix samples. Protein samples were left in the dark at room temperature for 1 h with vortex every 10 min. After an addition of 300 μL of 20% TCA solution, protein samples were incubated on ice for 15 min and centrifuged at 12,000 rpm for 5 min. Pellets were sequentially washed with 20% TCA solution and ethanol:ethyl acetate (1:1) solution to remove free DNPH. Pellets were then washed with 20% TCA twice and dried. Dried pellets were completely resuspended in 250 μL of 6 M guanidine hydrochloride. Carbonyl contents were determined using microplate reader (Molecular Devices) at a wavelength of 350 nm.

### 3.9. Preparation of Whole, Cytosolic, and Nuclear Proteins

To evaluate the nuclear translocation of Nrf2, we carried out the fractionation of nuclear and cytosolic proteins. After exposure to glutamate for 6 h, cells were harvested and lysed with cytoplasmic extraction buffer (HEPES 10 mM HEPES, pH 7.9, 10 mM KCl, 0.1 mM EDTA, 0.1% NP-40, and proteinase inhibitors). Lysate was centrifuged at 1200× *g* for 10 min, supernatants were used for cytosolic fraction. The pellets were washed with PBS three times and lysed with RIPA buffer (Cell Signaling, Danvers, MA, USA) containing freshly added with protease inhibitor cocktail (Roche, Indianapolis, IH, USA). For other signaling molecules, HT22 cells were lysed with RIPA buffer (Cell Signaling, Danvers, MA, USA) containing a protease inhibitor cocktail, 1 mM sodium orthovanadate (Na3VO4), and 1 mM sodium fluoride (NaF).

### 3.10. Western Blot Analysis

Equal amounts of proteins were separated using SDS-polyacrylamide gel electrophoresis and transferred to a polyvinylidene difluoride membrane (Merck Millipore, Darmstadt, Germany) [34]. To prevent the nonspecific binding of antibodies, the membranes were incubated with 5% skim milk in tris-buffered saline containing 0.1% Tween-20 (TBS-T). After washing three times with TPS-T, the membranes were then incubated with primary antibodies against extracellular signal-regulated kinase (ERK), phospho-ERK, p38, phospho-p38, JNK, phosphor-JNK, nuclear factor erythroid-derived 2-related factor 2 (Nrf2), heme oxygenase-1 (HO-1), and glyceraldehyde 3-phosphate dehydrogenase (GAPDH) for 1 h at room temperature. All primary antibodies were purchased from Cell Signaling. The membranes were then washed with TBS-T followed by incubation with appropriate horseradish peroxidase-conjugated secondary antibodies (Cell Signaling). Next, the membranes were reacted with SuperSignal West Femto Maximum Sensitivity Substrate (Thermo Scientific, Rockford, IL, USA), and immunoreactive bands were visualized using a Fusion Solo Chemiluminescence System (PEQLAB Biotechnologie GmbH, Erlangen, Germany). The optical densities of the immunoreactive bands were obtained using ImageJ software (Version 1.51J; National Institutes of Health, Bethesda, MD, USA) and normalized with those of the control. Data represented by fold-increases were compared to the control.

### 3.11. Statistical Analysis

All data described in this study were repeated at least three times and are represented as the mean ± S.E.M. Statistical significance was determined by one-way analysis of variance (ANOVA). Data were considered statistically significant at *p*-values less than 0.01.

## 4. Conclusions

Although the effect of PC-1 on various pathological conditions has been previously reported, our study demonstrated for the first time that PC-1 possesses strong neuroprotective effects against glutamate-induced apoptotic cell death in HT22 cells. In this study, we demonstrated that PC-1 possessed a strong antioxidant activity resulting in the inhibition of ROS accumulation and protein carbonylation. PC-1 also activates the Nrf2/HO-1 pathway as part of the defensive mechanism and inhibits the activation of MAPKs, including p38 and ERK. Our experimental results suggest that natural sources containing high levels of PC-1 are an interesting material for the therapeutic treatment of neurological disorders.
